# Low-Frequency and Rare-Coding Variation Contributes to Multiple Sclerosis Risk

**DOI:** 10.1016/j.cell.2018.09.049

**Published:** 2018-11-29

**Authors:** International Multiple Sclerosis Genetics Consortium, Mitja Mitrovič, Nikolaos A. Patsopoulos, Ashley H. Beecham, Theresa Dankowski, An Goris, Bénédicte Dubois, Marie B. D’hooghe, Robin Lemmens, Philip Van Damme, Helle Bach Søndergaard, Finn Sellebjerg, Per Soelberg Sorensen, Henrik Ullum, Lise W. Thørner, Thomas Werge, Janna Saarela, Isabelle Cournu-Rebeix, Vincent Damotte, Bertrand Fontaine, Lena Guillot-Noel, Mark Lathrop, Sandra Vukusik, Pierre-Antoine Gourraud, Till F.M. Andlauer, Viola Pongratz, Dorothea Buck, Christiane Gasperi, Antonios Bayas, Christoph Heesen, Tania Kümpfel, Ralf Linker, Friedemann Paul, Martin Stangel, Björn Tackenberg, Florian Then Bergh, Clemens Warnke, Heinz Wiendl, Brigitte Wildemann, Uwe Zettl, Ulf Ziemann, Hayrettin Tumani, Ralf Gold, Verena Grummel, Bernhard Hemmer, Benjamin Knier, Christina M. Lill, Felix Luessi, Efthimios Dardiotis, Cristina Agliardi, Nadia Barizzone, Elisabetta Mascia, Luisa Bernardinelli, Giancarlo Comi, Daniele Cusi, Federica Esposito, Laura Ferrè, Cristoforo Comi, Daniela Galimberti, Maurizio A. Leone, Melissa Sorosina, Julia Mescheriakova, Rogier Hintzen, Cornelia van Duijn, Charlotte E. Theunissen, Steffan D. Bos, Kjell-Morten Myhr, Elisabeth G. Celius, Benedicte A. Lie, Anne Spurkland, Manuel Comabella, Xavier Montalban, Lars Alfredsson, Pernilla Stridh, Jan Hillert, Maja Jagodic, Fredrik Piehl, Ilijas Jelčić, Roland Martin, Mireia Sospedra, Maria Ban, Clive Hawkins, Pirro Hysi, Seema Kalra, Fredrik Karpe, Jyoti Khadake, Genevieve Lachance, Matthew Neville, Adam Santaniello, Stacy J. Caillier, Peter A. Calabresi, Bruce A.C. Cree, Anne Cross, Mary F. Davis, Jonathan L. Haines, Paul I.W. de Bakker, Silvia Delgado, Marieme Dembele, Keith Edwards, Kathryn C. Fitzgerald, Hakon Hakonarson, Ioanna Konidari, Ellen Lathi, Clara P. Manrique, Margaret A. Pericak-Vance, Laura Piccio, Cathy Schaefer, Cristin McCabe, Howard Weiner, Jacqueline Goldstein, Tomas Olsson, Georgios Hadjigeorgiou, Bruce Taylor, Lotti Tajouri, Jac Charlesworth, David R. Booth, Hanne F. Harbo, Adrian J. Ivinson, Stephen L. Hauser, Alastair Compston, Graeme Stewart, Frauke Zipp, Lisa F. Barcellos, Sergio E. Baranzini, Filippo Martinelli-Boneschi, Sandra D’Alfonso, Andreas Ziegler, Annette Oturai, Jacob L. McCauley, Stephen J. Sawcer, Jorge R. Oksenberg, Philip L. De Jager, Ingrid Kockum, David A. Hafler, Chris Cotsapas

## Abstract

Multiple sclerosis is a complex neurological disease, with ∼20% of risk heritability attributable to common genetic variants, including >230 identified by genome-wide association studies. Multiple strands of evidence suggest that much of the remaining heritability is also due to additive effects of common variants rather than epistasis between these variants or mutations exclusive to individual families. Here, we show in 68,379 cases and controls that up to 5% of this heritability is explained by low-frequency variation in gene coding sequence. We identify four novel genes driving MS risk independently of common-variant signals, highlighting key pathogenic roles for regulatory T cell homeostasis and regulation, IFNγ biology, and NFκB signaling. As low-frequency variants do not show substantial linkage disequilibrium with other variants, and as coding variants are more interpretable and experimentally tractable than non-coding variation, our discoveries constitute a rich resource for dissecting the pathobiology of MS.

## Highlights

• Almost 20% of MS risk heritability can be attributed to common genetic variants
• We show that nearly 5% of heritability is explained by coding low-frequency variants
• We identify four novel genes driving risk independently of common-variant signals
• These genes would not have been found by common-variant studies

## Graphical Abstract

**Figure:**
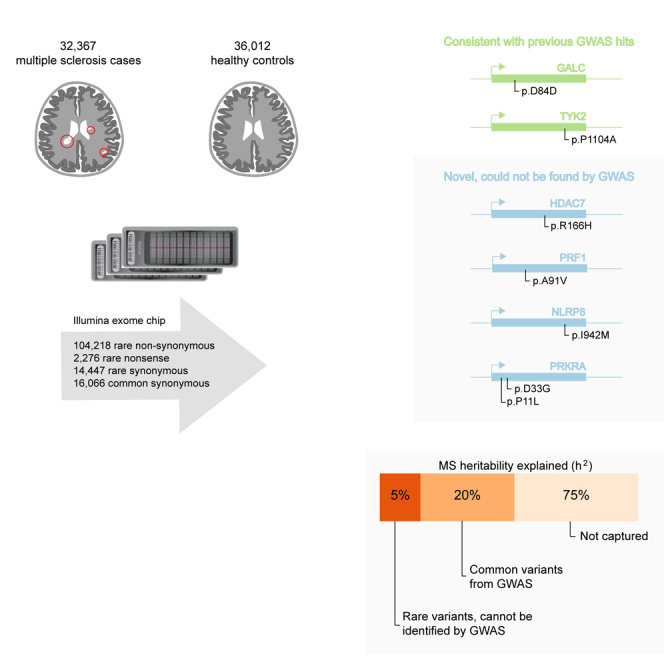

## Teaser

In a large multi-cohort study, unexplained heritability for multiple sclerosis is detected in low-frequency coding variants that are missed by GWAS analyses, further underscoring the role of immune genes in MS pathology.

## Introduction

Multiple sclerosis (MS; MIM 126200) is an autoimmune disease of the central nervous system and a common cause of neurologic disability in young adults (Compston and Coles, 2008). It is most prevalent in individuals of northern European ancestry and—in line with other complex, common disorders—shows substantial heritability (Binder et al., 2016), with a sibling standardized incidence ratio of 7:1 (Westerlind et al., 2014). Over the last 15 years, we have identified 233 independent, common-variant associations mediating disease risk by genome-wide association studies (GWASs) of increasing sample size (Andlauer et al., 2016, Australia and New Zealand Multiple Sclerosis Genetics Consortium (ANZgene), 2009, Baranzini et al., 2009, Beecham et al., 2013, De Jager et al., 2009, International Multiple Sclerosis Genetics Consortium et al., 2011, International Multiple Sclerosis Genetics Consortium et al., 2017, Jakkula et al., 2010, Martinelli-Boneschi et al., 2012, Nischwitz et al., 2010, Patsopoulos et al., 2011, Sanna et al., 2010, Burton et al., 2007). In our most recent meta-analysis of 14,802 MS cases and 26,703 controls, these effects—including 32 mapping to classical human leukocyte antigen (HLA) alleles and other variation in the major histocompatibility (MHC) locus (International Multiple Sclerosis Genetics Consortium et al., 2017, Moutsianas et al., 2015, Patsopoulos et al., 2013)—account for 7.5% of *h*^*2*^*g*, the heritability attributable to additive genetic effects captured by genotyping arrays, with a total of 19.2% of *h2g* attributable to all common variants in the autosomal genome (International Multiple Sclerosis Genetics Consortium et al., 2017). MS is thus a prototypical complex disease with a substantial portion of heritability determined by hundreds of common genetic variants, each of which explain only a small fraction of risk (Sawcer et al., 2014).

As with other common, complex diseases where large GWASs have been conducted, we find that although common variants (minor allele frequency [MAF] > 5%) account for the bulk of trait heritability, they cannot account for its entirety. Identifying the source of this unexplained heritability has thus become a major challenge (Manolio et al., 2009). Two hypotheses are frequently advanced: some common variants show epistatic (i.e., non-additive) interactions so that they contribute more risk in combination than each does alone, and a portion of risk is due to rare variants that cannot be imputed via linkage disequilibrium to common variants present on genotyping arrays and are therefore invisible to heritability calculations based on such arrays. The only evidence we have found for epistatic interactions between common MS risk variants is between two HLA haplotype families in the MHC locus (Moutsianas et al., 2015). This lack of epistatic interactions is consistent with other common, complex diseases, both of the immune system and beyond (Altshuler et al., 2008). We have also found no evidence that mutations in individual families drive disease risk in genome-wide linkage analyses of 730 MS families with multiple affected members (Sawcer et al., 2005). These results indicate that neither epistasis between known risk variants nor mutations in a limited number of loci are major sources of MS risk. They do not, however, preclude a role for variants present in the population at low frequencies, which cannot be imputed but are likely to individually contribute moderate risk.

**Figure S1:**
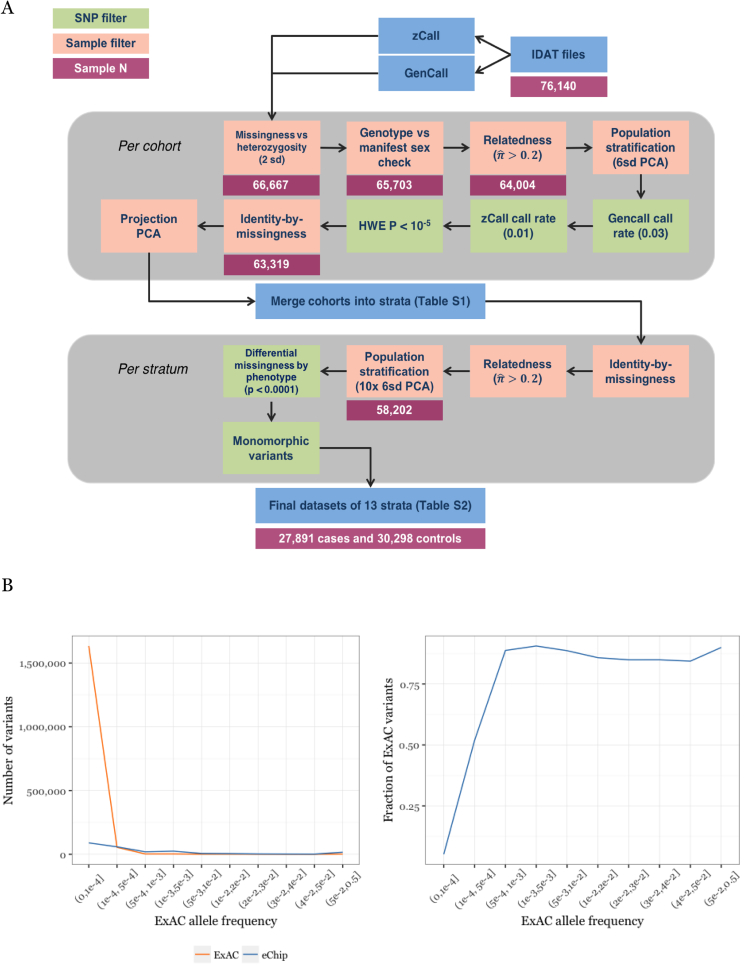
Data Quality Overview, Related to STAR Methods (A) QC process. We assembled 42 cohorts of data (either entire country-level collections or groups of samples processed as a batch; Table S1). We called common variant genotypes with the standard algorithm provided by Illumina (GenCall), and low-frequency variants with zCall, an algorithm specifically developed to call these variants on the exome chip (Goldstein et al., 2012). We performed initial quality control on each cohort separately to account for variation between batches and cohorts (upper gray region), then merged cohorts into 13 country-level strata. To ensure that these strata were uniform we then performed stringent quality control on each stratum (lower gray region) to produce our final dataset. (B) the exome chip captures a large fraction of ExAC (release version 1) low-frequency miss-sense variants. The exome chip captures the majority of variants present in ExAC (Lek et al., 2016) down to a minor allele frequency ∼0.0005, below which a large number of variants is observed (left). Thus, the overall coverage at very rare alleles (5 × 10^−4^ > MAF > 1.5 × 10^−5^, corresponding to a single allele seen in 33,370 non-Finnish European individuals in ExAC) is low (right).

Here, we report our assessment of the contribution of low-frequency variation in gene coding regions to MS risk. We conducted a meta-analysis of 120,991 low-frequency coding variants across all autosomal exons, including 104,218 non-synonymous and 2,276 nonsense variants, which are more likely to have a phenotypic effect. We analyzed a total of 32,367 MS cases and 36,012 controls drawn from centers across Australia, 10 European countries, and multiple US states, which we genotyped either on the Illumina HumanExome Beadchip (exome chip) or on a custom array (the MS chip), incorporating the exome chip content (International Multiple Sclerosis Genetics Consortium et al., 2017), and which satisfied our stringent quality control filters (Figure S1 and Table S1). The exome array is a cost-efficient alternative to exome sequencing, capturing approximately 88% of low-frequency and rare-coding variants present in 33,370 non-Finnish Europeans included in the Exome Aggregation Consortium (MAFs between 0.0001 and 0.05; Figure S1), and <5% of the extremely rare alleles present at even lower frequencies. Our study was well powered, with 80% power to detect modest effects at low frequency (odds ratio [OR] = 1.15 at MAF = 5%) and rare variants (OR = 1.5 at MAF = 0.5%) at a significance threshold of p < 3.5 × 10^−7^ (Bonferroni correction for the total number of variants genotyped).

## Results

**Figure 1:**
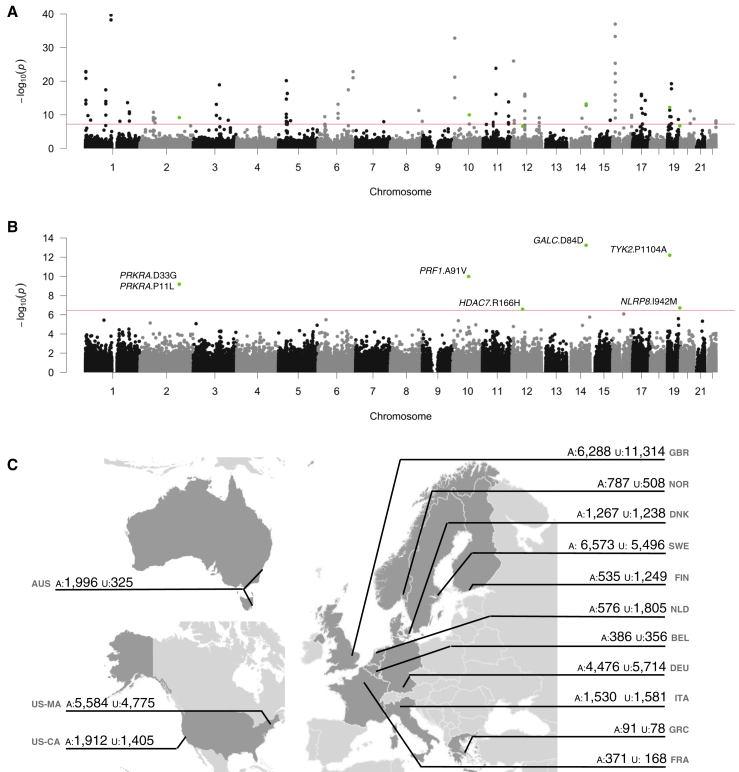
Rare-Coding Variants Are Associated to Multiple Sclerosis Risk in a Multi-cohort Study (A–C) We analyzed 120,991 low-frequency non-synonymous coding variants across all autosomal exons in 32,367 MS cases and 36,012 controls drawn across the International Multiple Sclerosis Genetics Consortium centers. We find evidence for association with both common variants with combined MAF > 5% (A) and with rare variants across the autosomes (B). We sourced samples from Australia, 10 European countries, and the United States (C). See also Figures S2 and S3.

**Figure S2:**
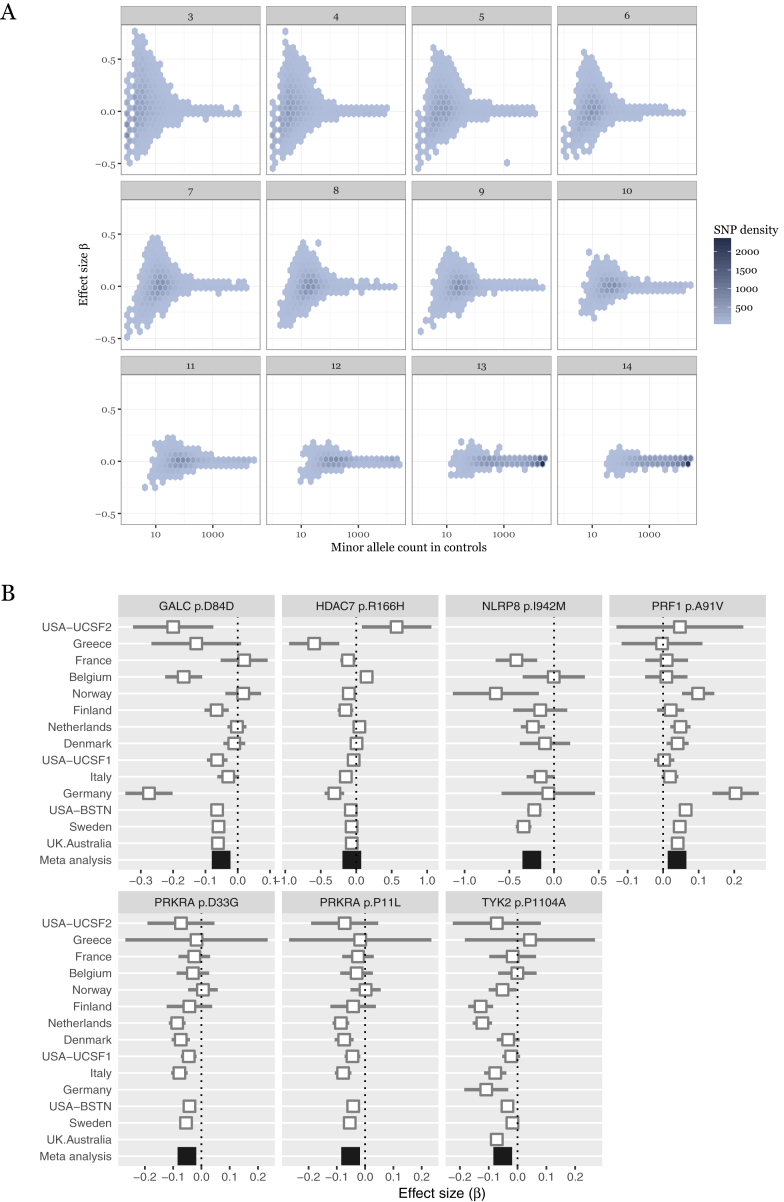
Low-Frequency Variant Association Statistic Characteristics, Related to Figure 1 (A) effect sizes increase at low minor allele frequency. We conducted a meta-analysis of 120,991 low-frequency coding variants across all autosomal exons, concentrating on non-synonymous variants which are more likely to have a phenotypic effect. We analyzed a total of 32,367 MS cases and 36,012 controls in thirteen strata. Here, we show that estimates of effect size (β or log odds ratio, y axis) increase at low allele frequency (number of minor alleles present in control samples, x axis). Because many low-frequency variants are not present in all cohorts, we stratify these data by number of cohorts in which a variant is polymorphic (subplots). Rarer variants have larger estimated effect sizes and are present in fewer cohorts. (B) forest plots for genome-wide significant low-frequency variants. Seven variants in six genes are significant in our analysis (p < *3.5 × 10*^*−7*^, Bonferroni correction for the total number of variants genotyped). Two of these (*TYK2* p.Pro1104Ala and *GALC* p.Asp84Asp), are in linkage disequilibrium with known GWAS hits. Studies are ordered by increasing sample size.

**Figure S3:**
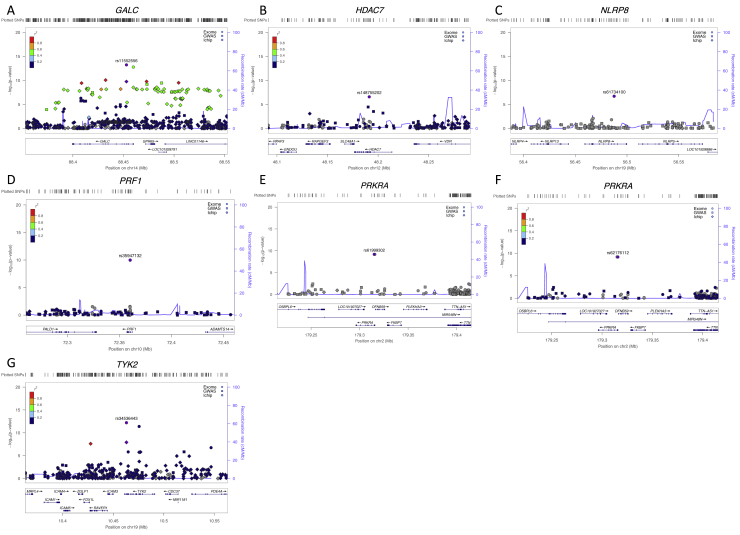
Patterns of Association for Common and Rare Variants in Seven Genome-wide Significant Loci, Related to Figure 1 Plots are centered on the seven variants reported in Figure 1 and Table 1. Each show LD and association signal of low frequency variants (circles, this study), and common variants from our most recent GWAS (squares, 14,802 MS cases and 26,703 controls; International Multiple Sclerosis Genetics Consortium et al., 2017) and the ImmunoChip meta-analysis (diamonds; Beecham et al., 2013). For *GALC* and *TYK2*, our most associated variants, rs11552556 and rs34536443 respectively, capture the common variant signals we have previously reported (panels A and G). For the remaining loci, our most associated variants show no LD to other variants, with no evidence of association in our common variant studies (panels B-F).

We first assessed the contribution of individual variants to MS risk by conducting a meta-analysis of association statistics across 14 country-level strata (Figure 1 and Table S1). We used linear mixed models to correct for population structure in 13 of these strata, estimated from the 16,066 common, synonymous coding variants present on the exome chip (i.e., variants with MAF > 5% in our samples). We included population structure-corrected summary statistics for the remaining cohort (from Germany), which has been previously described (Dankowski et al., 2015). As expected, we saw a strong correlation between effect size and variant frequency, with rarer alleles exerting larger effects (Figure S2). We found significant association between MS risk and seven low-frequency coding variants in six genes outside the extended MHC locus on chromosome 6 (Table 1 and Figure S3). Two of these variants (*TYK2* p.Pro1104Ala, overall MAF 4.1% in our samples; *GALC* p.Asp84Asp, overall MAF 3.9%) are in regions identified by our latest MS GWAS and show linkage disequilibrium with the common-variant associations we have previously reported (International Multiple Sclerosis Genetics Consortium et al., 2011). The remaining associations are novel, with the variants neither in linkage disequilibrium nor physical proximity to common variant association signals and thus not imputable in our GWASs (Table S2).

**Table 1:** Coding Variants Associated to Multiple Sclerosis Risk

Chr	Position	rsID	Minor Allele	MAF	Studies Observed	P Value	OR	LCI	UCI	Gene	AA Change
14	88452945	rs11552556	A	3.9%	14	5.759E−14	0.95	0.93	0.97	GALC	Synonymous D84D
19	10463118	rs34536443	G	4.1%	13	6.282E−13	0.95	0.93	0.97	TYK2	Missense P1104A
10	72360387	rs35947132	A	5.0%	14	1.043E−10	1.04	1.02	1.06	PRF1	Missense A91V
2	179315031	rs61999302	T	5.6%	12	6.467E−10	0.95	0.93	0.97	PRKRA	Missense D33G
2	179315726	rs62176112	A	5.6%	12	6.633E−10	0.95	0.93	0.97	PRKRA	Missense P11L
19	56487619	rs61734100	C	0.2%	9	1.925E−07	0.78	0.67	0.91	NLRP8	Missense I942M
12	48191247	rs148755202	T	1.4%	14	2.597E−07	0.94	0.91	0.98	HDAC7	Missense R166H

We analyzed 120,991 low-frequency non-synonymous coding variants across all autosomal exons in 32,367 MS cases and 36,012 controls drawn from centers across Australia, 10 European countries, and multiple US states. Genome positions are relative to hg19. The two variants in *PRKRA* are in linkage disequilibrium (R^2^ = 1, D` = 1 in the 1000 Genomes European samples). These variants lie in common variant risk loci found in our previous GWAS (International Multiple Sclerosis Genetics Consortium et al., 2017).

We were struck by the observation that the minor allele is protective in six of the seven cases in Table 1, a trend we also observe at less stringent significant thresholds (Figure S2). This pattern is unusual in common-variant studies: for example, in our most recent GWAS, 101/200 non-MHC effects showed that the minor allele increases risk. To test if this phenomenon is due to our strata containing more cases than controls, we randomly resampled 4,000 affected and 4,000 unaffected samples in our three largest strata and calculated association statistics as for our main analysis. In this symmetric design, we found no bias toward protective minor alleles at even modest levels of significance (Table S3). Thus, low-frequency variants do not preferentially decrease MS risk rather than increase it.

**Figure 2:**
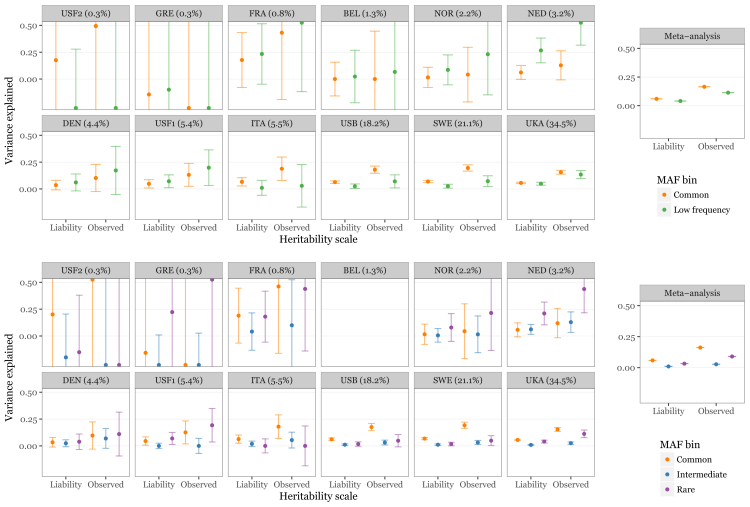
Rare Variants Explain a Substantial Portion of Multiple Sclerosis Heritability We estimated the MS risk heritability explained by common variants (MAF > 5%) and low-frequency non-synonymous coding variation (MAF < 5%) in each of 13 cohorts genotyped on the exome chip using genome-wide complex trait analysis (GCTA; top). By meta-analyzing these estimates across cohorts, we found that low-frequency variants explain 11.34% of heritability on the observed scale, which corresponds to 4.1% on the liability scale (right top). After dividing the low-frequency variants into intermediate (5% > MAF > 1%) and rare (MAF < 1%; bottom), we found that the latter alone explains 9.0% heritability on the observed scale (3.2% on the liability scale; bottom right). Meta-analysis confidence intervals are small and visually occluded by the mean estimate plot characters. Cohorts (abbreviations as in Table S1) are ordered by sample size, with the percentage of the overall sample size shown in each subplot title. We could not obtain estimates for either model for our Finnish cohort (see STAR Methods; not shown), or for the three-component model for our Belgian cohort (bottom, top row, fourth from left). Both cohorts are small, which may explain the failure to converge.

Though we are able to identify individual low-frequency variants associated with MS risk, we recognize that we cannot detect all such variants at genome-wide significance, even in a study of this magnitude. We thus sought to quantify the overall contribution of low-frequency coding variation to MS risk. We used a restricted maximum-likelihood approach to model heritability attributable to genotypic variation across the genome that was initially developed for common-variant analyses (Yang et al., 2011) and later shown to also perform well for rare variants, as in the present case (Mancuso et al., 2016). In each of the 13 strata that comprise our data, we estimated the proportion of heritability explained by common (MAF > 5%) and low-frequency (MAF ≤ 5%; Table S4) variants on the exome arrays (Yang et al., 2011). We included genotype-derived principal components to further control for population stratification. By meta-analyzing these estimates across the twelve strata where the restricted maximum likelihood model converged, we found that low-frequency variants explain 11.34% (95% confidence interval 11.33%–11.35%) of the observed difference between cases and controls (mean estimate 4.1% on the liability scale; Figure 2). We further partitioned the low-frequency variants into intermediate (5% > MAF ≥ 1%) and rare (MAF < 1%) and found that the latter alone explain 9.0% (95% confidence interval 8.9%–9.1%) on the observed scale (mean estimate 3.2% on the liability scale; Figure 2). We note that six of the eight genome-wide significant variants presented in Table 1 are of intermediate frequency and thus are not included in the rare category. We capture the majority, though not all, of known common risk variants to some extent with the common variants on the exome chip (Table S5); our analysis therefore adequately, though imperfectly, models this portion of the frequency spectrum. Our results thus indicate that many more non-synonymous rare variants contribute to MS risk but are not individually detectable at genome-wide thresholds, even in large studies like ours.

In this study, we show that low-frequency coding variation explains a fraction of MS risk that cannot be attributed to common variants across the genome. We capture most, but not all, low-frequency missense variants (Figure S1), suggesting our heritability estimates for low-frequency and rare variation are conservative. This broadly agrees with previous reports that such variants contribute to complex traits, including Alzheimer’s disease (Sims et al., 2017) and schizophrenia (Purcell et al., 2014), where heritability modeling similar to ours supports a role for rare variants. Studies of quantitative phenotypes shared by the entire population, such as height (Marouli et al., 2017), serum lipid levels (Liu et al., 2017), and blood cell traits (Chami et al., 2016, CHARGE Consortium Hematology Working Group, 2016) have also reported novel associations to low-frequency coding variants outside the large number of known GWAS loci in each trait. However, a meta-analysis of different type 2 diabetes study designs found no associations outside common-variant GWAS regions (Fuchsberger et al., 2016), though this may be due to the heterogeneity of sample ascertainment and study design. In aggregate, therefore, our results and these past studies demonstrate that rare coding variants contribute a fraction of common, complex trait heritability. These results also agree with both theoretical expectation and empirical observations that low-frequency coding variants are under natural selection and are unlikely to increase in frequency in the population (Nelson et al., 2012, Schoech et al., 2017, Zeng et al., 2018). Thus, some portion of disease-associated variants, and hence the genes they influence, may not be detectable with conventional GWAS designs.

The newly discovered genes have clear immunological functions, confirming that MS pathogenesis is primarily driven by immune dysfunction. The associated polymorphisms show negligible linkage disequilibrium with other variants (Table S2), so the genes harboring them are likely to be relevant to disease. *PRF1* encodes perforin, a key component of the granzyme-mediated cytotoxicity pathways used by several lymphocyte populations. In addition to cytotoxic lymphocytes and natural killer (NK) cells (House et al., 2015), perforin-dependent cytotoxicity is also seen in CD4^+^FOXP3^+^ regulatory T cells (Tregs), which show aberrant, T helper-like IFNγ secretion in MS patients (Dominguez-Villar et al., 2011). The MS risk variant rs35947132 (p.Ala91Val) is associated with a decrease in target cell-killing efficiency and increases in IFNγ secretion by NK cells (House et al., 2015), which aligns with the aberrant Treg phenotype observed in MS. This decreased cytotoxicity efficiency will prolong average cell-cell interactions with target cells, and such extended interactions are known to increase T cell-receptor-mediated signaling and induce changes to T cell phenotypes, especially secretion of IFNγ and other cytokines (Constant et al., 1995). Similarly, *HDAC7* encodes the class II histone deacetylase 7, which potentiates the repressive effects of *FOXP3*, the master regulator governing naive CD4^+^ T cell development into Tregs (Bettini et al., 2012, Li et al., 2007). It also regulates T cell survival during their development in the thymus (Kasler et al., 2011). *PRKRA* encodes protein kinase interferon-inducible double-stranded RNA-dependent activator; in response to double-stranded RNA due to virus infection, it heterodimerizes with protein kinase R to inhibit EIF2a-dependent translation, resulting in upregulation of nuclear factor κB (NFκB) signaling, interferon production, and eventually, apoptosis (Sadler and Williams, 2008). NFκB-mediated signaling is a core feature of MS pathogenesis, which we have shown to be altered by at least one MS-associated variant (Housley et al., 2015) and may be the relevant mechanism for this gene. Finally, *NLRP8* is an intracellular cytosolic receptor active in innate immune responses; the Ile942Met MS risk variant rs61734100 is detected only in individuals with European ancestry in ExAC, consistent with the higher prevalence of MS in European ancestry populations.

## Discussion

Broadly, therefore, our results show that low-frequency genetic variation explains a portion of MS risk and that this variation impacts genes not detectable by common-variant association studies. Our heritability modeling demonstrates that more low-frequency and rare-variant associations remain to be discovered, though larger sample sizes will be required to increase statistical power. Recent attention has focused on changes to the adaptive immune system as pathogenic for MS, particularly to functional changes in helper T cell subsets and B cells after they have been released from the thymus and bone marrow, respectively, into the peripheral blood stream. These processes remain important to pathogenesis and are supported by a wealth of data, including our own GWAS (International Multiple Sclerosis Genetics Consortium et al., 2017). However, two of the four new genes we report (*PRKRA* and *NLRP8*) have clear functions in innate immunity, and HDAC7 plays a central role in the development of T cells in the thymus. Roles for both innate immune function and thymic development in MS pathogenesis are also supported by pathway analyses of our most recent GWAS data (International Multiple Sclerosis Genetics Consortium et al., 2017), an independent observation due to the lack of linkage disequilibrium (LD) between the variants in this study and those in our GWAS and the non-overlapping sample collections. Our data thus expand the scope of immune function relevant to MS pathogenesis.

The mechanisms whereby our newly discovered variants alter MS risk will require detailed experimental dissection: even when we can directly implicate specific genes and variants, these can have diverse consequences across multiple cell types. For example, perforin 1 has key—and potentially distinct—roles in cytotoxic T cells, regulatory helper T cells, NK cells, and other cell types. Both the effects of the variant on each of these functions and their relevance to MS pathogenesis will thus require demonstration, as is the case for the genes central to IFNγ biology, Treg function, and the NFκB signaling pathway.

## STAR★Methods

### Key Resources Table

**Table:** 

REAGENT or RESOURCE	SOURCE	IDENTIFIER
**Deposited Data**		

Genotype data	This paper (Not all data are available at EGA—please contact dac@imsgc.net for access to the entire dataset)	EGAS00001003195

**Software and Algorithms**		

Plink v1.9	Purcell et al., 2007	https://www.cog-genomics.org/plink/1.9/
GCTA	Yang et al., 2011	http://cnsgenomics.com/software/gcta
EIGENSOFT	Price et al., 2006	https://github.com/DReichLab/EIG
The R Project for Statistical Computing	R Development Core Team, 2017	https://www.R-project.org
QC and analysis pipeline	This paper	https://github.com/cotsapaslab/IMSGCexomechip

### Contact for Reagent and Resource Sharing

Further information and requests for resources and reagents should be directed to and will be fulfilled by the Lead Contact, Chris Cotsapas (chris.cotsapas@yale.edu).

### Experimental Model and Subject Details

We assembled a total of 76,140 samples (36,219 cases, 38,629 controls and 1,292 samples with missing phenotype information) from across the International MS Genetics Consortium (IMSGC; Table S1). All individuals gave informed consent at enrolment, and recruitment was monitored by research ethics boards in *Australia*: University of Tasmania; Bond University; University of Sydney. *Belgium*: Katholieke Universiteit, Leuven. *Canada*: McGill University, Montreal. *Denmark*: University of Copenhagen. *Finland*: University of Helsinki. *France*: Hôpital Pitié-Salpêtrière, Paris; Hôpital Neurologique Pierre Wertheimer, Bron; Université de Nantes. *Germany*: University of Lübeck; Max Planck Institute of Psychiatry, Munich; Technische Universität München; Johannes Gutenberg University-Medical Center, Mainz; Klinikums at Augsburg, Hanover and Großhadern Munich; Universitätsklinikums of Hamburg, Erlangen, Gießen/Marburg, Leipzig, Köln, Münster, Heidelberg, Rostock, and Tübingen, the Universität Ulm. *Greece*: University of Larissa. *Italy*: University of Eastern Piedmont, Novara; Ospedale Maggiore, Novara; San Raffaele Scientific Institute, Milan; University of Milan. *Netherlands*: Erasmus MC, Rotterdam; VU University Medical Center, Amsterdam. *Norway*: University of Bergen; University of Oslo. *Spain*: Universitat Autònoma de Barcelona. *Sweden*: Karolinska Institutet, Stockholm. *Switzerland*: University Hospital Zurich. *United States of America*: Yale University, New Haven CT; Brigham & Women’s Hospital, Boston MA; the University of Miami, Miami FL; UCSF and USB San Francisco, CA; Kaiser Permanente Divison of Research, Oakland, CA; Johns Hopkins University Baltimore MD; Washington University St Louis, St Louis MO; Vanderbilt University Medical Center, Nashville TN; Brigham Young University, Provo, UT; Case Western Reserve University, Cleveland, OH; The University of Pennsylvania and the Children’s Hospital of Philadelphia, PA; Columbia University Medical Center, New York, NY. *United Kingdom*: MRC Biostatistics Unit, Cambridge; University of Cambridge; Keele University; King’s College London; University of Oxford; and University College London.

### Method Details

We genotyped these either on the Illumina HumanExome Beadchip (exome chip) or on a previously described custom array (International Multiple Sclerosis Genetics Consortium et al., 2017) including the exome chip content, both manufactured by Illumina Inc. We called genotypes both with Illumina’s default algorithm, gencall, and zCall, specifically developed to call low-frequency variants where all three groups of genotypes may not be observed (Goldstein et al., 2012).

An overview of our quality control process is shown in Figure S1; we used PLINK (Purcell et al., 2007) for all analyses unless otherwise noted. Briefly, we first excluded samples with low genotyping rate, extreme heterozygosity rate, inconsistent genotypic and recorded sex; we also removed closely related samples, keeping the relative with least missing data. Next, we removed population outliers by calculating genotype principal components using 16,066 common variants in linkage disequilibrium (r^2^ < 0.1) across the exome. We used EIGENSOFT 6 (Price et al., 2006) and FlashPCA (Abraham and Inouye, 2014) for cohorts with more than 10.000 individuals. We next removed variants with > 3% gencall missing data rate for variants with minor allele frequency MAF > 5%, or > 1% zCall missing data rate for variants with MAF < 5%. We also removed variants out of Hardy-Weinberg equilibrium (p < 10^−5^). Next, we removed samples with high similarity in missing genotypes (“identity by missingness”) indicative of production artifact, and samples with missing phenotype information. Finally, we again removed any remaining population outliers using projection principal component analysis. We calculated 30 principal components for 1,092 individuals in 1000 Genomes reference populations, again using the 16,066 common variants in linkage disequilibrium (r^2^ < 0.1) across the exome. We then projected the IMSGC samples into this space and excluded individuals more than six standard deviations from loading means as previously described (Price et al., 2006). We performed the projection and outlier detection and removal steps a total ten times to gradually remove more subtle population outliers.

We compiled cases and controls into strata for analysis as shown in Table S1. In total, we removed 17,938/76,140 (24%) samples either due to low data quality or as population outliers, leaving a final dataset of 27,891 cases and 30,298 controls in 13 strata (Figure S1 and Table S1). Separately, we included summary statistics from 4,476 MS cases and 5,714 controls from Germany, genotyped on the exome chip as previously described (Dankowski et al., 2015), giving us a total of 32,367 MS cases and 36,012 controls for analysis.

### Quantification and Statistical Analysis

#### Exome chip coverage of ExAC variants

To assess how thoroughly the exome chip assesses low-frequency coding variation genome-wide, we compared it to the list of variants reported by the Exome Aggregation Consortium, ExAC (Lek et al., 2016), in their data release version 1. We filtered their summary table of all ExAC variants (available at ftp://ftp.broadinstitute.org/pub/ExAC_release/release1/manuscript_data/ExAC.r1.sites.vep.table.gz and last accessed 15 November 2017) for nonsynonymous coding variants passing their quality control, with at least one minor allele observed in non-Finnish European samples. We identified which of these variants are represented on the exome chip by comparing genomic coordinates.

#### Univariate association analysis

We used mixed linear models for association analysis, as implemented in GCTA (Yang et al., 2011). In each of our 13 genotype-level strata, we calculated genetic relatedness matrices from 16,066 common, noncoding variants (overall MAF > 0.05) in linkage equilibrium (all pairwise r^2^ < 0.1) present on the exome chip, and with these calculated univariate association statistics for each autosomal variant present on the exome chip. To further control for population stratification, we also calculated genotypic principal components with the 16,066 common variants, and included these as covariates to the association analysis. We also included genotypic sex and chip type as covariates. We combined statistics across strata using inverse-variance-weighted meta-analysis, also as implemented in GCTA (Yang et al., 2011). As the bulk of exome chip variants are not common and do not show appreciable linkage disequilibrium, we controlled for multiple tests with a Bonferroni correction for the number of low-frequency variants, to give a genome-wide significance threshold of p < 3.58 × 10e^-7^ (0.05/139,764 variants with a combined MAF < 0.05 in controls and a heterogeneity index I^2^ < 50 in our meta-analysis).

#### Heritability estimation

We used GCTA to calculate the heritability attributable to groups of variants in each of our 13 genotype-level strata (Yang et al., 2011). In each stratum, we ran two sets of models: a two-component model, estimating the heritability attributable to common and low-frequency (MAF ≤ 0.05) variants; and a three component model with rare (MAF ≤ 0.01), intermediate (0.01 < MAF ≤ 0.05), and common variants. In all strata, common variants are the set of 16,066 independent variants (overall MAF > 0.05) used for population stratification calculations in the univariate analysis above. We computed genetic relatedness matrices for each component of each model, then calculated narrow-sense heritability (*h*^*2*^) with 100 iterations of constrained restricted maximum likelihood (REML) fitting, assuming a disease prevalence of 0.001. We also included the principal components of population structure computed for the univariate analysis as covariates. As anticipated, several of the smaller cohorts presented fitting issues: no models converged for FIN; both three-component and two-component fits for UCSF2, and the three-component model for GRE would not converge under constraint and so were run without constraints; and the three-component model for BEL converged on two exactly equally likely solutions after 10,000 iterations. For the latter, we chose the most conservative estimates of variance explained. We combined these estimates with inverse variance-weighted meta-analysis.

### Data and Software Availability

Meta-analysis summary statistics are available at http://imsgc.net/. Due to varying privacy laws across countries, some of our genotype data are available from the European Genome-phenome Archive (deposited under accession EGAS00001003195), with the remainder available directly from participating centers. A single request for all data access may be submitted to the IMSGC Data Access Committee (dac@imsgc.net). Our QC and analysis pipeline is available at https://github.com/cotsapaslab/IMSGCexomechip.

## Supplemental Information

Document S1. Tables S1–S4

Table S5. Imputation Quality of 186 Most Significant GWAS Variants, Related to Table 1 We used our scaffold of 16,066 common, independent variants to impute each of the 200 common MS risk variants reported in our latest GWAS (International Multiple Sclerosis Genetics Consortium et al., 2017), in each of our three largest cohorts. We can impute 162, 156, and 162 variants in Sweden, UK/Australia and USA-Boston with an INFO score > = 0.25; these numbers drop to 72, 67, and 75 of 200 at INFO > = 0.5 and 22, 20, and 20 at INFO > = 0.75, respectively.
