# Low-Frequency and Rare-Coding Variation Contributes to Multiple Sclerosis Risk

**DOI:** 10.1016/j.cell.2019.06.016

**Published:** 2019-06-27

**Authors:** International Multiple Sclerosis Genetics Consortium, Mitja Mitrovič, Nikolaos A. Patsopoulos, Ashley H. Beecham, Theresa Dankowski, An Goris, Bénédicte Dubois, Marie B. D’hooghe, Robin Lemmens, Philip Van Damme, Helle Bach Søndergaard, Finn Sellebjerg, Per Soelberg Sorensen, Henrik Ullum, Lise W. Thørner, Thomas Werge, Janna Saarela, Isabelle Cournu-Rebeix, Vincent Damotte, Bertrand Fontaine, Lena Guillot-Noel, Mark Lathrop, Sandra Vukusik, Pierre-Antoine Gourraud, Till F.M. Andlauer, Viola Pongratz, Dorothea Buck, Christiane Gasperi, Antonios Bayas, Christoph Heesen, Tania Kümpfel, Ralf Linker, Friedemann Paul, Martin Stangel, Björn Tackenberg, Florian Then Bergh, Clemens Warnke, Heinz Wiendl, Brigitte Wildemann, Uwe Zettl, Ulf Ziemann, Hayrettin Tumani, Ralf Gold, Verena Grummel, Bernhard Hemmer, Benjamin Knier, Christina M. Lill, Felix Luessi, Efthimios Dardiotis, Cristina Agliardi, Nadia Barizzone, Elisabetta Mascia, Luisa Bernardinelli, Giancarlo Comi, Daniele Cusi, Federica Esposito, Laura Ferrè, Cristoforo Comi, Daniela Galimberti, Maurizio A. Leone, Melissa Sorosina, Julia Mescheriakova, Rogier Hintzen, Cornelia van Duijn, Charlotte E. Theunissen, Steffan D. Bos, Kjell-Morten Myhr, Elisabeth G. Celius, Benedicte A. Lie, Anne Spurkland, Manuel Comabella, Xavier Montalban, Lars Alfredsson, Pernilla Stridh, Jan Hillert, Maja Jagodic, Fredrik Piehl, Ilijas Jelčić, Roland Martin, Mireia Sospedra, Maria Ban, Clive Hawkins, Pirro Hysi, Seema Kalra, Fredrik Karpe, Jyoti Khadake, Genevieve Lachance, Matthew Neville, Adam Santaniello, Stacy J. Caillier, Peter A. Calabresi, Bruce A.C. Cree, Anne Cross, Mary F. Davis, Jonathan L. Haines, Paul I.W. de Bakker, Silvia Delgado, Marieme Dembele, Keith Edwards, Kathryn C. Fitzgerald, Hakon Hakonarson, Ioanna Konidari, Ellen Lathi, Clara P. Manrique, Margaret A. Pericak-Vance, Laura Piccio, Cathy Schaefer, Cristin McCabe, Howard Weiner, Jacqueline Goldstein, Tomas Olsson, Georgios Hadjigeorgiou, Bruce Taylor, Lotti Tajouri, Jac Charlesworth, David R. Booth, Hanne F. Harbo, Adrian J. Ivinson, Stephen L. Hauser, Alastair Compston, Graeme Stewart, Frauke Zipp, Lisa F. Barcellos, Sergio E. Baranzini, Filippo Martinelli-Boneschi, Sandra D’Alfonso, Andreas Ziegler, Annette Oturai, Jacob L. McCauley, Stephen J. Sawcer, Jorge R. Oksenberg, Philip L. De Jager, Ingrid Kockum, David A. Hafler, Chris Cotsapas

(Cell *175*, 1679–1687.e1–e7; November 29, 2018)

We the editors of Cell have been informed by the Lead Contact and corresponding author of this paper, Dr. Chris Cotsapas, that further analyses following the publication of the paper have indicated that two variants (rs61999302 encoding PRKRA.D33G and rs62176112 encoding PRKRA.P11L) reported in this paper to be associated with MS risk were in fact spurious associations due to the presence of a dispersed duplication event, in which this region of chromosome 2 is duplicated in the MHC region of chromosome 6. The potential for this dispersed duplication to lead to spurious associations has been previously documented (Wellcome Trust Case Control Consortium et al., 2010). Upon learning of the potential problems associated with signals in this region, the authors used the data reported in this paper to impute classical HLA alleles and amino acid variants using SNP2HLA (Jia et al. 2013) and found that the two reported variants in PRKRA showed extensive linkage disequilibrium with genotyped and imputed HLA variants, including known MS risk alleles. Conditioning on any of these known variants was sufficient to explain the reported association, leading to the conclusion that these two signals are driven by the duplication and are not true associations of the reported variants with MS risk. With this note, we and the authors would like to alert readers to this issue. The remaining findings in the paper are unchanged, as are the main conclusions of the paper. The authors would also like to thank Dr. Matthew Hurles (Wellcome Sanger Institute) for bringing this issue to their attention.

Furthermore, in the printed version of the experimental model and subject details section of the STAR methods, Köln is listed as one of the patient recruitment centers, but Düsseldorf should have been listed instead. One of the authors, Dr. Clemens Warnke, although currently based in Köln, in fact contributed patient samples collected in Düsseldorf to the study. This has been corrected in the article online, and the authors apologize for any confusion these issues may have caused.
