# Low-Frequency and Rare-Coding Variation Contributes to Multiple Sclerosis Risk

**DOI:** 10.1016/j.cell.2020.01.002

**Published:** 2020-01-23

**Authors:** International Multiple Sclerosis Genetics Consortium, Mitja Mitrovič, Nikolaos A. Patsopoulos, Ashley H. Beecham, Theresa Dankowski, An Goris, Bénédicte Dubois, Marie B. D’hooghe, Robin Lemmens, Philip Van Damme, Helle Bach Søndergaard, Finn Sellebjerg, Per Soelberg Sorensen, Henrik Ullum, Lise W. Thørner, Thomas Werge, Janna Saarela, Isabelle Cournu-Rebeix, Vincent Damotte, Bertrand Fontaine, Lena Guillot-Noel, Mark Lathrop, Sandra Vukusik, Pierre-Antoine Gourraud, Till F.M. Andlauer, Viola Pongratz, Dorothea Buck, Christiane Gasperi, Antonios Bayas, Christoph Heesen, Tania Kümpfel, Ralf Linker, Friedemann Paul, Martin Stangel, Björn Tackenberg, Florian Then Bergh, Clemens Warnke, Heinz Wiendl, Brigitte Wildemann, Uwe Zettl, Ulf Ziemann, Hayrettin Tumani, Ralf Gold, Verena Grummel, Bernhard Hemmer, Benjamin Knier, Christina M. Lill, Felix Luessi, Efthimios Dardiotis, Cristina Agliardi, Nadia Barizzone, Elisabetta Mascia, Luisa Bernardinelli, Giancarlo Comi, Daniele Cusi, Federica Esposito, Laura Ferrè, Cristoforo Comi, Daniela Galimberti, Maurizio A. Leone, Melissa Sorosina, Julia Mescheriakova, Rogier Hintzen, Cornelia van Duijn, Charlotte E. Teunissen, Steffan D. Bos, Kjell-Morten Myhr, Elisabeth G. Celius, Benedicte A. Lie, Anne Spurkland, Manuel Comabella, Xavier Montalban, Lars Alfredsson, Pernilla Stridh, Jan Hillert, Maja Jagodic, Fredrik Piehl, Ilijas Jelčić, Roland Martin, Mireia Sospedra, Maria Ban, Clive Hawkins, Pirro Hysi, Seema Kalra, Fredrik Karpe, Jyoti Khadake, Genevieve Lachance, Matthew Neville, Adam Santaniello, Stacy J. Caillier, Peter A. Calabresi, Bruce A.C. Cree, Anne Cross, Mary F. Davis, Jonathan L. Haines, Paul I.W. de Bakker, Silvia Delgado, Marieme Dembele, Keith Edwards, Kathryn C. Fitzgerald, Hakon Hakonarson, Ioanna Konidari, Ellen Lathi, Clara P. Manrique, Margaret A. Pericak-Vance, Laura Piccio, Cathy Schaefer, Cristin McCabe, Howard Weiner, Jacqueline Goldstein, Tomas Olsson, Georgios Hadjigeorgiou, Bruce Taylor, Lotti Tajouri, Jac Charlesworth, David R. Booth, Hanne F. Harbo, Adrian J. Ivinson, Stephen L. Hauser, Alastair Compston, Graeme Stewart, Frauke Zipp, Lisa F. Barcellos, Sergio E. Baranzini, Filippo Martinelli-Boneschi, Sandra D’Alfonso, Andreas Ziegler, Annette Oturai, Jacob L. McCauley, Stephen J. Sawcer, Jorge R. Oksenberg, Philip L. De Jager, Ingrid Kockum, David A. Hafler, Chris Cotsapas

(Cell *175*, 1679–1687.e1–e7; November 29, 2018)

It has come to our attention that in preparing the final version of this article, the authors inadvertently misspelled the last name of author Charlotte E. Teunissen as “Charlotte E. Theunissen.” This error has been corrected in the article online.

In the Editorial Note (Cell *178*, 262, June 27, 2019), the editors refer to the original version of the published manuscript. That version contained a misspelled name, and as that has now been corrected, we are updating the Editorial Note as well.

