# Supplementary material for: Low-Frequency and Rare-Coding Variation Contributes to Multiple Sclerosis Risk
Source: Cell. 2018 Nov 29;175(6):1679–1687.e7. doi: 10.1016/j.cell.2018.09.049 (PMC6269166; doi:10.1016/j.cell.2018.09.049)
Supplement: Document S1. Tables S1–S4 [file mmc1.pdf]

Cell, Volume *175*

Supplemental Information

Low-Frequency and Rare-Coding Variation Contributes to Multiple Sclerosis Risk

International Multiple Sclerosis Genetics Consortium

SUPPLEMENTAL TABLES AND LEGENDS

Stratum	Chip version	Cohort	Pre-QC samples	Post-QC samples	Case/Con	Male/Female	Polymorphic variants	Rare variants (MAF < 5%)
Belgium	Exome_v1.0	BEL	397	386	386/0/0	123/263/0	78,044	52,806
	Exome_v1.1	BEL	499	356	0/356/0	168/188/0	78,044	52,806
Denmark	Exome_v1.0	DEN	552	487	302/185/0	175/312/0	97,122	72,397
	MS_chip	DEN	2,019	1,732	888/844/0	724/1,008/0	97,122	72,397
	MS_chip	DEN	329	286	77/209/0	135/151/0	97,122	72,397
Finland	Exome_v1.0	FIN	558	535	535/0/0	153/382/0	74,570	48,724
	Exome_v1.0	FIN	1,699	1,249	0/1249/0	540/709/0	74,570	48,724
France	Exome_v1.0	FRA	624	539	371/168/0	184/355/0	80,502	53,909
Germany	Exome_v1.0	GER	10,190	10,190	4,476/5,714	N/A	N/A	N/A
Greece	MS_chip	GRE	195	169	91/78/0	58/111/0	56,384	29,567
Italy	Exome_v1.0	ITA	964	799	798/1/0	303/496/0	118,504	93,746
	Exome_v1.1	ITA	939	805	0/805/0	559/246/0	118,504	93,746
	MS_chip	ITA	1,956	1,507	732/775/0	726/781/0	118,504	93,746
Netherlands	Exome_v1.0	NED	504	400	400/0/0	115/285/0	127,220	102,830
	Exome_v1.1	NED	2,181	1,805	0/1,805/0	810/995/0	127,220	102,830
	Exome_v1.0	NED	218	176	176/0/0	65/111/0	127,220	102,830
Norway	Exome_v1.0	NOR	891	839	648/191/0	249/590/0	82,749	57,846
	Exome_v1.0	NOR	342	317	0/317/0	163/154/0	82,749	57,846
	MS_chip	NED-NOR	533	139	139/0/0	37/102/0	82,749	57,846
Sweden	Exome_v1.0	SWE	1,139	968	509/459/0	263/705/0	127,220	102,830
	MS_chip	SWE	3,030	1,972	1,099/873/0	494/1,478/0	127,220	102,830
	MS_chip	SWE	2,993	2,000	1,147/853/0	498/1,502/0	127,220	102,830
	MS_chip	SWE	3,151	2,081	1,164/917/0	541/1,540/0	127,220	102,830
	MS_chip	SWE	3,067	2,059	1,192/867/0	488/1,571/0	127,220	102,830
	MS_chip	SWE	3,061	2,023	1,099/924/0	507/1,516/0	127,220	102,830
	MS_chip	SWE	1,235	966	363/603/0	258/708/0	127,220	102,830
UK/Australia	Exome_v1.0	AUS	1,776	1,408	1,408/0/0	333/1,075/0	141,707	119,407
	Exome_v1.0	AUS	333	172	172/0/0	31/141/0	141,707	119,407
	Exome_v1.0	UK	6,400	5,316	0/5316/0	2,934/2,382/0	141,707	119,407
	Exome_v1.0	UK	1,700	1,449	1,449/0/0	428/1,021/0	141,707	119,407
	Exome_v1.0	UK	126	111	111/0/0	37/74/0	141,707	119,407
	Exome_v1.0	UK	1,038	861	0/861/0	493/368/0	141,707	119,407
	Exome_v1.0	UK	156	140	140/0/0	37/103/0	141,707	119,407
	MS_chip	AUS	1,060	741	416/325/0	181/560/0	141,707	119,407
	MS_chip	UK	2,666	1,992	956/1,036/0	700/1,292/0	141,707	119,407
	MS_chip	UK	4,400	3,552	1,699/1,853/0	618/2,934/0	141,707	119,407
	MS_chip	UK	1,886	1,616	747/869/0	385/1,231/0	141,707	119,407
	MS_chip	UK	3,036	2,565	1,186/1,379/0	934/1,631/0	141,707	119,407
US-BSTN	Exome_v1.0	US	2,442	2,065	1,102/963/0	554/1,511/0	150,886	126,830
	Exome_v1.0	US	129	87	87/0/0	23/64/0	150,886	126,830
	MS_chip	US	4,542	3,486	1,847/1,639/0	1,026/2,460/0	150,886	126,830
	MS_chip	US	3,510	2,330	1,215/1,115/0	741/1,589/0	150,886	126,830
	MS_chip	US	3,032	2,391	1,333/1,058/0	699/1,692/0	150,886	126,830
US-UCSF1	Exome_v1.0	US	1,169	940	558/382/0	300/640/0	128,704	102,898
	Exome_v1.0	US	1,971	1,792	1,019/773/0	287/1,505/0	128,704	102,898
	Exome_v1.0	US	1,670	580	330/250/0	212/368/0	128,704	102,898
US-UCSF2[a]	/	/	/	/	/	/	53,363	27,257
**Total**	**/**	**/**	**76,140**	**68,379**	**32,367/36,012**	**N/A**	**/**	**/**

[a]Strata US-UCSF1 and US-UCSF2 consist of individuals from the same three US cohorts.

Table S1 – genotype-level samples included in our study. 58,189/76,140 (76.5%) of our samples passed quality control and could be assigned to one of thirteen strata. 9,473/17,951 (52.8%) of failed samples did not pass a heterogeneity versus missing data rate filter, suggesting either poor data quality or population stratification (detailed in Figure S1). We used two versions of Illumina's HumanCore Exome array: the standard product (version 1.0; designated *Exome_v1.x* in the chip type column) and a customized version including ~100,000 additional variants we specified (designated *MS_chip*), described elsewhere (Patsopoulos et al., 2017). Belgian control samples were genotyped at the Center for Inherited Disease Research (CIDR, Baltimore, MD, USA) on the Illumina 5M array (Illumina, San Diego, CA, USA) as part of the Stroke Genetics Network (SiGN). Data for

10,190 samples from Germany were received as post-QC summary statistics and are not included in Table S1. This gave us a total of 68,379 samples in our analysis. We had very few Australian control samples and so merged them with samples from the UK.

Gene	*GALC*	*TYK2*	*PRF1*	*PRKRA*	*PRKRA*	*NLRP8*	*HDAC7*
Exome chip significant variants	rs11552556	rs34536443	rs35947132	rs61999302	rs62176112	rs61734100	rs148755202
Chr	14	19	10	2	2	19	12
Position	88,452,945	10,463,118	72,360,387	179,315,031	179,315,726	56,487,619	48,191,247
Closest replicated IMSGC GWAS hit	rs116899835	rs34536443	rs17741873	rs6738544	rs6738544	rs1465697	rs701006
r2	0.841	1	0.003	0	0	na	na
D'	0.943	1	0.133	0.061	0.005	na	na
Distance (bp)	395,801	0	3,293,413	12,674,325	12,673,630	6,650,373	9,915,589
GWAS cohort							
ANZ	0.8301	0.7688	0.1668	0.0154	0.0181	na	na
AUS1	0.7906	0.7498	0.2505	0.0313	0.0326	na	na
BWH_MIGEN	0.8127	0.1791	0.3835	0.0188	0.0233	na	na
Berkeley	0.867	0.774	0.3059	0.0241	0.0272	na	na
CE1	0.79	0.776	0.2899	0.036	0.0334	na	na
FINLAND	0.9376	0.7586	0.3775	0.0229	0.0228	na	na
GeneMSA_DU	0.8626	0.8481	0.4339	0.0192	0.0189	na	na
GeneMSA_SW	0.7705	0.7953	0.3058	0.0289	0.026	na	na
GeneMSA_US	0.8064	0.7121	0.3742	0.0192	0.0197	na	na
IMSGC	0.7428	0.1241	0.2075	0.0147	0.0196	na	na
MEDI7	0.7513	0.7304	0.4008	0.0227	0.0206	na	na
UK	0.8041	0.7796	0.2684	0.0347	0.0341	na	na
US2	0.802	0.7444	0.2834	0.0363	0.0365	na	na
VIKINGS	0.8424	0.7933	0.398	0.0254	0.0263	na	na
Rotterdam	0.8024	0.8093	0.2137	0.0237	0.0278	na	na
**Median**	**0.8041**	**0.7688**	**0.3058**	**0.0237**	**0.026**	**na**	**na**
Minimum	0.7428	0.1241	0.1668	0.0147	0.0181	na	na
Maximum	0.9376	0.8481	0.4339	0.0363	0.0365	na	na

Table S2 – Five of seven exome-wide significant variants show no linkage disequilibrium with common MS risk variants and cannot be imputed from common variants. To establish if our low-frequency variants are capturing common variant associations, we identified the closest common variant signal in our latest GWAS of 14,802 MS cases and 26,703 controls (IMSGC, 2017). As expected, the variants in *GALC* and *TYK2* capture previously known associations. The other five variants are >1Mb from the closest signal and show negligible LD levels to common MS risk variants. These thus represent novel associations and cannot be capturing common variant signals. We have previously imputed ~8.6 million 1000 Genomes variants in 14,802 MS cases and 26,703 controls from our GWAS, none of which overlap the samples presented in this study (IMSGC, 2017). In these samples, we are able to impute the *GALC* and *TYK2* variants with some certainty but cannot do so for the five remaining variants. These thus represent novel associations and cannot be capturing common variant signals. The association patterns in each locus shown graphically in Figures S5-S11.

P-value threshold for selecting variants	MAF range	Stratum	Number of variants	Effect direction		
				OR > 1	OR < 1	Binomial p
[1,0]	[1,0.05)	Sweden	22,398	11,404	10,994	0.006
		UK/Australia	20,515	10,355	10,160	0.176
		US-BSTN	22,026	11,203	10,823	0.011
	[0.05,0.01)	Sweden	8,464	4,252	4,212	0.672
		UK/Australia	7,534	3,853	3,681	0.049
		US-BSTN	7,970	4,015	3,955	0.509
	[0.01,0]	Sweden	84,526	42,514	42,012	0.085
		UK/Australia	90,687	46,062	44,625	1.86E-06
		US-BSTN	108,763	56,719	52,044	1.31E-45
[0.05,0]	[1,0.05)	Sweden	1,234	615	619	0.932
		UK/Australia	1,185	616	569	0.181
		US-BSTN	1,296	628	668	0.279
	[0.05,0.01)	Sweden	460	243	217	0.244
		UK/Australia	412	223	189	0.104
		US-BSTN	464	226	238	0.610
	[0.01,0]	Sweden	2,654	1,333	1,321	0.831
		UK/Australia	2,674	1,378	1,296	0.117
		US-BSTN	3,449	1,702	1,747	0.454
[1E-4,0]	[1,0.05)	Sweden	21	10	11	1
		UK/Australia	34	15	19	0.608
		US-BSTN	14	4	10	0.180
	[0.05,0.01)	Sweden	0	-	-	na
		UK/Australia	2	1	1	1
		US-BSTN	2	1	1	1
	[0.01,0]	Sweden	3	3	-	0.250
		UK/Australia	3	3	-	0.250
		US-BSTN	1	-	1	1
[1E-6, 0]	[1,0.05)	Sweden	5	3	2	1
		UK/Australia	5	-	5	0.063
		US-BSTN	0	-	-	na
	[0.05,0.01)	Sweden	0	-	-	na
		UK/Australia	1	1	-	1
		US-BSTN	0	-	-	na
	[0.01,0]	Sweden	0	-	-	na
		UK/Australia	0	-	-	na
		US-BSTN	0	-	-	na

Table S3 – downsampling to an equal number of cases and controls balances out the occurrence of risk and protective effects in low-frequency and rare variants. We were struck by the observation that the minor allele is protective in six of the seven cases in Table 1, a trend we also observe at less stringent significant thresholds (Figure S3). To test if this phenomenon is due to our strata containing more cases than controls, we randomly resampled 4,000 affected and 4,000 unaffected samples in our three largest strata and calculated association statistics as for our main analysis. In this symmetric design, we found no bias towards protective minor alleles at even modest levels of significance.

Stratum	Number of cases	Number of controls	Number of SNPs in heritability calculations			
			Common	Intermediate	Rare	Low-frequency
			MAF > 0.05	0.05 > MAF > 0.01	MAF < 0.01	MAF < 0.05
Belgium	386	356	16,059	8,763	42,088	50,851
Denmark	1,267	1,238	16,059	8,298	61,425	69,723
Finland	535	1,249	16,059	9,535	37,192	46,727
France	371	168	16,059	9,103	42,778	51,881
Greece	91	78	16,059	8,640	19,516	28,156
Italy	1,530	1,581	16,059	7,802	82,841	90,643
Netherlands	576	1,805	16,059	8,494	61,699	70,193
Norway	787	508	16,059	8,641	47,025	55,666
Sweden	6,573	5,496	16,059	8,308	90,833	99,141
UK/Australia	8,284	11,639	16,059	7,388	108,050	115,438
US-BSTN	5,584	4,775	16,059	7,780	114,642	122,422
US-UCSF1	1,815	1,330	16,059	8,463	90,929	99,392
US-UCSF2	92	75	16,059	9,958	15,962	25,920

Table S4 – Number of variants in heritability calculations for each MAF category. In each of the thirteen strata that comprise our data, we estimated the proportion of heritability explained by common, low-frequency and rare variants. We calculated MAF in each stratum independently. Note that, as expected, we see more rare variants in larger strata.

Table S5 – Imputation quality of 186 most significant GWAS variants *(Table S5 is saved separately in the file "Imputation quality of 186 most significant GWAS variants.xlsx")*. We used our scaffold of 16,066 common, independent variants to impute each of the 200 common MS risk variants reported in our latest GWAS (Patsopoulos *et al*, 2017), in each of our three largest cohorts. We can impute 162, 156, and 162 variants in Sweden, UK/Australia and USA-Boston with an INFO score >= 0.25; these numbers drop to 72, 67, and 75 of 200 at INFO >= 0.5 and 22, 20, and 20 at INFO >= 0.75, respectively.
